# Impaired post-stroke collateral circulation in sickle cell anemia mice

**DOI:** 10.3389/fneur.2023.1215876

**Published:** 2023-09-26

**Authors:** Emily J. Bian, Ching-Wen Chen, Chih-Mei Cheng, Chia-Yi Kuan, Yu-Yo Sun

**Affiliations:** ^1^Department of Neuroscience, Center for Brain Immunology and Glia (BIG), University of Virginia School of Medicine, Charlottesville, VA, United States; ^2^Department of Immunology, Duke University School of Medicine, Durham, NC, United States; ^3^Department of Biomedical Science and Environmental Biology, Kaohsiung Medical University, Kaohsiung City, Taiwan; ^4^Department of Medical Research, Kaohsiung Medical University, Kaohsiung City, Taiwan; ^5^Institute of Biopharmaceutical Sciences, National Sun Yat-sen University, Kaohsiung City, Taiwan

**Keywords:** penumbra, photothrombosis, laser speckle contrast imaging, collateral circulation, sickle cell anemia

## Abstract

Patients with sickle cell anemia (SCA) have a high incidence of ischemic stroke, but are usually excluded from thrombolytic therapy due to concerns for cerebral hemorrhage. Maladaptation to cerebral ischemia may also contribute to the stroke propensity in SCA. Here we compared post-stroke cortical collateral circulation in transgenic sickle (SS) mice, bone marrow grafting-derived SS-chimera, and wildtype (AA) controls, because collateral circulation is a critical factor for cell survival within the ischemic penumbra. Further, it has been shown that SS mice develop poorer neo-collateral perfusion after limb ischemia. We used the middle cerebral artery (MCA)-targeted photothrombosis model in this study, since it is better tolerated by SS mice and creates a clear infarct core versus peri-infarct area. Compared to AA mice, SS mice showed enlarged infarction and lesser endothelial proliferation after photothrombosis. SS-chimera showed anemia, hypoxia-induced erythrocyte sickling, and attenuated recovery of blood flow in the ipsilateral cortex after photothrombosis. In AA chimera, cerebral blood flow in the border area between MCA and the anterior cerebral artery (ACA) and posterior cerebral artery (PCA) trees improved from 44% of contralateral level after stroke to 78% at 7 d recovery. In contrast, blood flow in the MCA-ACA and MCA-PCA border areas only increased from 35 to 43% at 7 d post-stroke in SS chimera. These findings suggest deficits of post-stroke collateral circulation in SCA. Better understanding of the underpinnings may suggest novel stroke therapies for SCA patients.

## Introduction

The collateral circulation plays a critical role in the outcomes of cerebral ischemia. This is because when a large cerebral artery is occluded, it produces an ischemic core where neurons perish rapidly due to energy depletion and a surrounding ischemic penumbra where neurons are paralyzed but remain salvageable, as long as the “oligemia times duration” distress level is below a threshold (1–3). The core/penumbra concept highlights the importance of early thrombolysis therapy in ischemic stroke, as captured by the maxim “time is brain.” Yet, the roles of collateral circulation in salvaging the ischemic penumbra are often under-appreciated.

**Figure 1:**
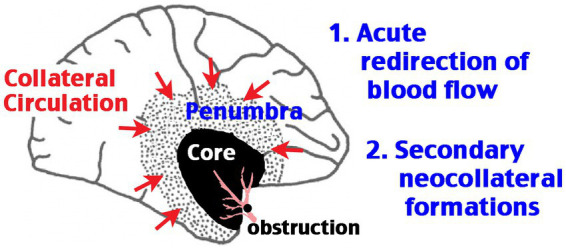
Schematic of the two phases of post-stroke cerebral collateral circulation. Obstruction of a large cerebral artery produces an ischemic core with severely diminished blood flow and a rim of ischemic penumbra, where the blood flow is reduced, but remaining above the threshold of infarction. The core/penumbra distinction is dynamic, as the survival of tissue in the penumbra area depends on large-vessel recanalization or the extent of collateral circulation. The first phase of collateral circulation involves redirection of blood flow in the anastomosis vascular network acutely after occlusion. The second phase of collateral circulation requires the formation of neo-collateral blood vessels.

Cerebral collateral circulation refers to the ancillary vasculature network, primarily the leptomeningeal anastomosis, that supplements cerebral blood flow if the primary conduits fail. Animal studies suggested two phases of post-stroke collateral circulation: acute redirection of blood flow toward the ischemic area and secondary neo-collateral vessel formations [Figure 1, modified from Faber et al. (4)]. Live imaging of cortical surface vessels indicates robust redistribution of collateral blood flow after occlusion of cerebral arteries, which may involve the endothelial nitric oxide synthase (eNOS) and the sphingosine 1-phospahte (S1P) signaling pathways, followed by remodeling of the existing collaterals and the formation of neo-collateral vessels, particularly in chronic obstructive conditions (5–7). Clinical trials indicate that strong collateral circulation at the time of acute stoke imaging predicts good outcomes for either thrombolytic or thrombectomy therapy (8, 9). In contrast, several risk factors for poorer stroke outcomes, including diabetes and aging, are associated with diminished collateral leptomeningeal flow (10, 11). These correlations suggest that the greater collateral circulation, the longer and more likely the ischemic brain tissue remains viable. This concept is epitomized by the newer maxim, “time is brain, but collaterals set the pace” (12).

Stroke is a severe and common complication of sickle cell anemia (SCA), a systemic genetic disorder due to the hemoglobin “S” mutation that causes erythrocytes to become sticky and sickle-shaped under hypoxia or dehydration, leading to hemolytic anemia and inflammatory or proliferative-occlusive vasculopathy (13). Without frequent blood transfusions, 11% of the SCA children encounter large overt stroke before age 20, and 37% of the SCA children develop silent cerebral infarct and mild cognition impairment by age 14 (14, 15). Even at acute ischemic stroke, the SCA patients are generally excluded from fibrinolytic therapies owing to concerns for induced cerebral hemorrhage. The mouse models of SCA recapitulate the stroke propensity and inferior outcomes in SCA patients (16–18). In addition to erythrocyte sickling and endothelial dysfunction, deficits in post-stroke collateral circulation may be another critical factor for higher stroke propensity and poor outcomes in SCA patients. Consistent with this notion, Townes sickle (SS) mice showed marked reduction in neo-collateral formations after ligation of the femoral artery (19). Consequently, SS mice manifested a higher incidence of foot necrosis than wildtype mice (80% vs. 15%) and more often required amputation (25% vs. 5%) (19). In view of these observations, it is tempting to speculate that SS mice may have poorer collateral circulation after stroke, but this scenario has not been tested to date. Thus, the goal of this study is to test whether transgenic and SS chimera mice display deficits in post-stroke cortical collateral circulation and neo-angiogenesis. If this scenario is confirmed, to improve collateral circulation may be a useful therapeutic strategy for SCA patients and/or general population.

To this end, we compared the infarct size and endothelial cell proliferation between SS and AA mice after MCA-targeted photothrombosis (20, 21). We chose this model as it induces a clear infarct core versus peri-infarct area and is better tolerable by SS mice than more invasive stroke models, such as the intraluminal suture MCA-occlusion and transient hypoxia-ischemia (17). We used laser speckle contrast imaging (LSCI) to compare the recovery of blood flow in ipsilateral cortex post-photothrombosis between SS and AA chimera for 7 days (22). Our results suggest deficits of post-stroke collateral circulation and neo-angiogenesis in SCA, which may be promising targets for acute stroke treatment.

## Materials and methods

### Animals

Humanized sickle cell Townes (Tg-SS) mice (Jax No. 013071 [16]), wildtype mice (C57BL/6), and CD45 congenic mice (CD45.1 positive leukocytes, Jax No. 002014) were obtained from the Jackson Laboratory. Mice were housed under pathogen-free conditions and fed on the standard laboratory chow *ad libitum*. Only male mice, aged at 2–6 months, were used for experiemnts. All mice were cared for in agreement with the guidelines approved by the Institutional Animal Care and Use Committee (IACUC) at the University of Virginia.

### Bone marrow transplantation

In addition to Townes SS mice obtained directly from the Jackson Laboratory, bone marrow SS chimeras were used in this study in order to obtain a larger number of age-matched male SS and AA chimera for comparison. These chimera mice were generated with slight modifications from the previous report (23). Briefly, 5–10 million bone marrow cells isolated from donor mice were intravenously injected to two-month old x-ray irradiated (with two doses of 5.5 Gy given 4 h apart) recipient mice. With this number of bone marrow cell grafting, one Townes SS mouse was used to create six SS chimera mice (i.e., in a 1:6 ratio of yield). All donor and recipient mice were male and obtained from the Jackson Laboratory.

In total, three cohorts of SS and AA chimeras were generated for the presnet study. For use as controls, non-sickle chimera (AA chimera) mice were also produced by transplanting bone marrow cells from CD45 congenic mice (CD45.1 positive leukocytes) into irradiated wildtype C57BL/6 animals (CD45.2 positive leukocytes) in a 1:6 ratio. To evaluate the chimerism in SS chimera, bone marrow cells from Tg-SS mice (CD45.2^+^) were transplanted to irradiated CD45.1^+^ wildtype mice. At 8 weeks after bone marrow grafting, mice were tested for chemerism via flow cytometry (Attune NxT) of peripheral blood and complete blood count analysis (Abaxis HM5 Vetscan Hematology Analyzer). Of note, the HM5 Vetscan Hematology Analyzer does not provide reticulocyte counts.

### Flow cytometry

Heparin-treated peripheral mouse blood was treated with anti-CD16/32 antibody (2.4G2, BD Biosciences) to reduce nonspecific binding against mouse CD4 (RM4-5, Biolegend), CD8 (53–6.7, BD-Biosciences), CD11b (M1/70, BD Biosciences), CD45.1 (A20, Biolegend), CD45.2 (104, Biolegend), and Ly6C (HK1.4, Biolegend). Samples were analyzed using the Attune NxT Flow Cytometer equipped with 405 nm, 488 nm, 561 nm, and 640 nm lasers. Compensation was performed with isotype controls and single antibody-stained samples, and the data were analyzed using the FlowJo software (version 10.8.1).

### Middle cerebral artery-targeted Photothrombosis

The male mice were subjected to MCA-targeted photothrombosis, as described (20, 21). Brifely, mice were anesthetized by 2% isoflurane in an induction chamber and then secured in a supine position to a surgical stage, while anesthesia was maintained under 1–1.5% isoflurane via a nose cone. The left common carotid artery was ligated using 6–0 silk suture, and the skin of the neck was closed with monofilament suture. Next, to target the MCA primary branch, the mouse was flipped to a prone position, and both the scalp and temporalis muscle were retracted to expose the edge of parietal and squamosal bones to visualize the proximal MCA branching point. A drill (Rotex 782, Dentamerica) was used to create a 1 mm diameter and 0.2 mm-deep impression in the squamosal bone above the proximal MCA branching point, taking care to keep the skull and dura intact. 50 mg/kg Rose Bengal (Sigma-Aldrich) was then injected into the retro-orbital sinus and allowed to perfuse through the body for 5 min before photoactivation was introduced. A 543 nm laser beam (5 mW, Melles Griot) of around 1 mm in diameter was applied to the skull-drilled site from 2 inches away. Wearing laser protection goggles, one can observe the distal MCA branches initially showing red fluoresence under photoactivation, but the signal disapeared after around 10 min, indicating succesful cessation of the MCA blood flow. After 20 min of illumination, the wound was closed with suture, and the mouse recovered on a heated pad. All procedures were approved by the IACUC at University of Virginia.

### TTC staining

The infarct volume after MCA-targeted photothrombosis was assessed using the *ex vivo* 2,3,5-Triphenyltetrazolium Chloride (TTC, Sigma-Aldrich) stain. Mice first underwent transcardial perfusion with 10 mL PBS, and the brains were removed, covered with 3–5% agar, and sectioned into 1 mm slices by a Vibratome (Stoelting No. 51425). The fresh brain slices were stained with TTC solution (2% in PBS) until color change to red was observed. The images were obtained by digital microscope, and the infarct volume was quantified using ImageJ software (NIH).

### Evans blue and EdU injections

To identify microvascular leakage, 200 μL 2% Evans Blue (EB, Sigma-Aldrich) dye was injected through the tail vein at 7 d after MCA-photothrombosis. To label the cells enaged in post-stroke mitosis, 10 mg/ kg EdU (Sigma-Aldrich) was injected intraperitoneally daily to mice from day 0 to day 2 after MCA-photothrombosis. At 7 d post-stroke, mice was anesthetized and underwent transcardiac perfusion of PBS and 4% paraformaledhyde. The mouse brains were harvested and sectioned into 20 μm slices on cryostat. Brain sections were immunostained using a commercial kit (Click-iT Plus EdU cell proliferation kit, Invitrogen) and Isolectin B4 staining (I21413, Invitrogen; 1:100 dilution) to visualize endothelial cells on epifluorescence microscopy (BX43, Olympus). Double labelled cells were quantified using the ImageJ software (NIH).

### Laser speckle contrast imaging

Cerebral blood flow (CBF) fluctuations were monitored by laser speckle contrast imaging system (MoorFLPI-2; Moor Instruments), as previously described (24, 25). At the indicated time after MCA-photothrombosis, mouse was anesthetized and placed in a prone position with an incision made along the scalp midline to expose the skull. Craniotomy is not necessary for laser speckle contrast imaging in mice, which maintains the dura integrity (and the intracranial pressure) and facilitates multiple longitudinal measurments of post-stroke CBF recovery. CBF was recorded in both cortical hemipsheres at the designated times for at least 5 min after signal stabilization were reached. Every reported datum was the average of blood flow signals over 5 min in each mouse. The CBF was shown using a 250 color palette of perfusion units and quantified as the percentage to the corresponding contralateral region.

### Statistical analysis

Statistical analysis was performed by GraphPad Prism using Mann–Whitney test. The *p*-values less than 0.05 were considered a significant difference. All values were expressed as mean ± SEM.

## Results

### Townes SS mice exhibit larger infarction and poorer neo-angiogenesis after focal cerebral ischemia

**Figure 2:**
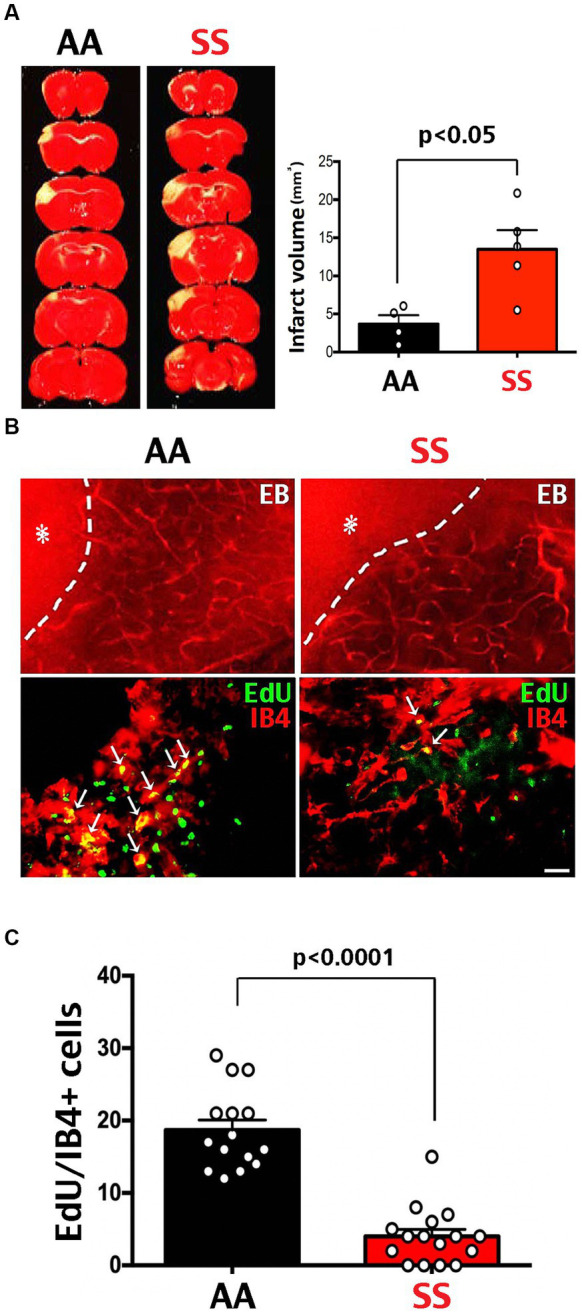
Larger infarction and poorer neo-angiogenesis in Townes sickle mice. **(A)** TTC stain at 3 days after MCA-targeted photothrombosis showed a larger infarct size in SS (*n* = 5) than AA mice (*n* = 4). Shown are the mean and SEM, with *p*-values determined by unpaired *t*-test. **(B)** Tail vein injection of Evans blue (EB) dye and Isolectin B4 (IB4)/EdU double-labeling on day 7 after photothrombosis in transgenic SS and AA mice. The extravasation of EB dye (asterisk) defines the ischemic core, with the dash line denoting the infarction border. Scale bar: 200 μm in EB dye images, and 20 μm in IB4/EdU double-labeling. **(C)** Co-localized EdU/IB4 immuno-signals within the 500 μm peri-infarct area were counted as cells per section to compare endothelial proliferation after stroke, since EdU was injected daily from day 0 to 2 after MCA-stroke. Fewer IB4/EdU colocalized cells were found in the peri-infarct area in SS mice compared with AA mice (*p* < 0.0001 by Mann–Whitney test, *n* = 15 sections in three AA-chimera mice; *n* = 16 sections in three SS-chimera mice).

MCA-targeted photothrombosis was used to compare post-stroke recovery in Townes SS and wildtype (AA) mice. Compared to AA mice, SS mice showed larger infarct volumes at 24 h after MCA-photothrombosis (Figure 2A). Evans Blue (EB) dye was applied intravenously at 7 d post-stroke to compare cerebral vasculature in the per-infarct area between SS and AA mice. Because EB binds to albumin and is not metabolically active, the presence of EB-albumin indicates blood vessels, and its leakage into brain parenchyma denotes extravasation and delineates the ischemic core (asterisks in Figure 2B). Intraperitoneal injection of EdU from day 0 to 2 post-stroke was used to detect endothelial cell proliferation. Compared to AA mice, SS mice showed fewer Isolectin B4 (IB4)/EdU double-positive cells in the peri-infarct area at 7 d after photothrombosis (Figures 2B,C). These results suggest reduced post-stroke neo-angiogenesis in Townes SS mice.

### SS-chimera mice show anemia, splenomegaly, and erythrocyte-sickling under hypoxia

**Figure 3:**
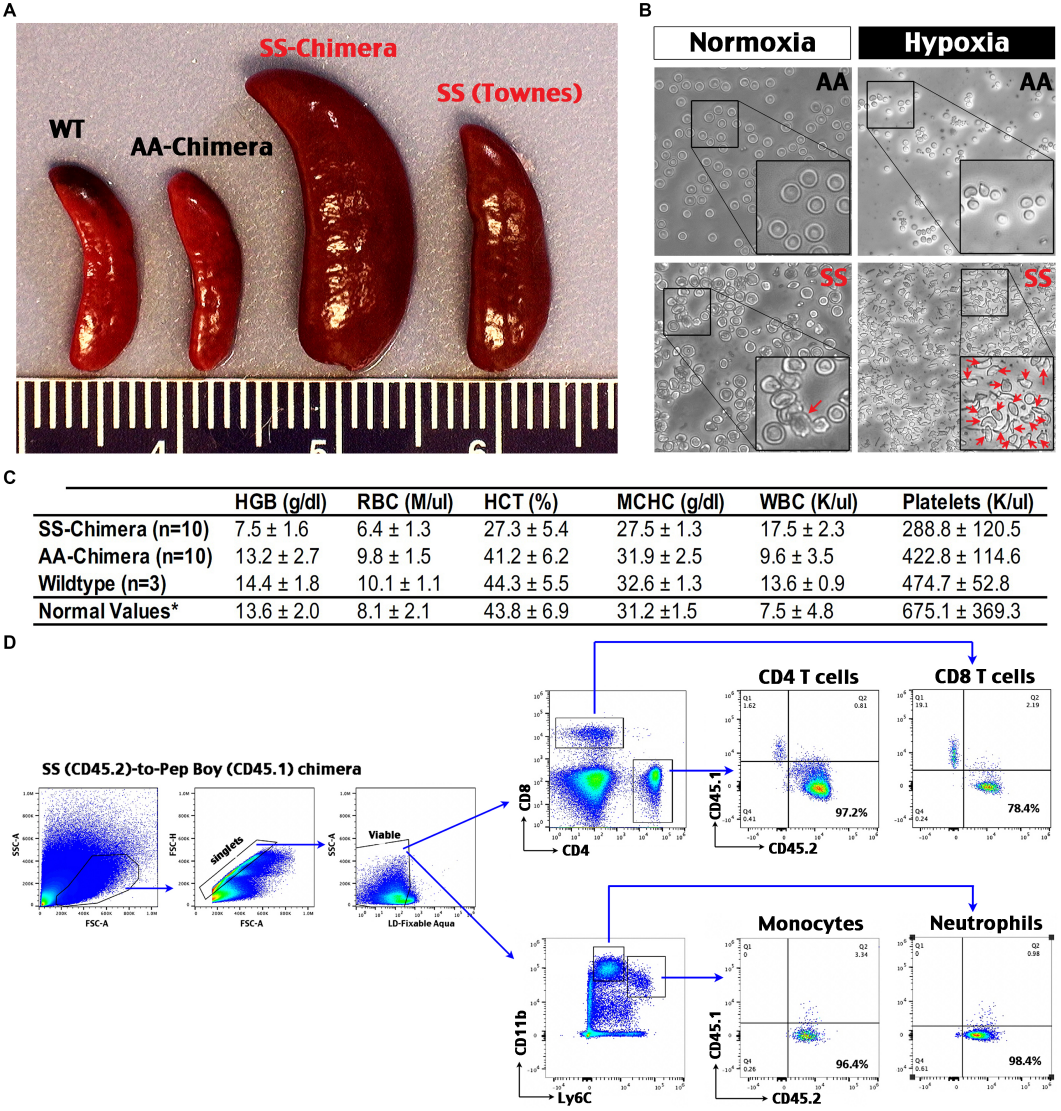
Hematological phenotypes and high chimerism and in SS-chimera mice. **(A)** Representative images of the spleens from wildtype (WT), Townes SS mouse, AA- and SS-chimera at 2 months after bone marrow transplantation (BMT). Note splenomegaly in Townes SS and SS-chimera mice. **(B)** After incubation in hypoxia (1% O_2_) for 1 h, nearly all RBCs in SS-chimera deformed into a sickle-shaped morphology, while RBCs in AA-chimera resist severe deformity even after exposure to the same hypoxic insult. **(C)** Complete blood count (CBC) of peripheral blood showed anemia in SS-chimera (*n* = 10), as in a reduction of hemoglobin (HGB), RBC counts, hematocrit (HCT), the mean corpuscular hemoglobin concentration (MCHC), and platelet count (PLT) compared with AA-chimera (*n* = 10) and WT (*n* = 3) mice. SS-chimera also showed a small increase of white blood cells (WBC) compared with AA-chimera. ^*^The normal values of mouse hematology are based on the Johns Hopkins Phenotyping Core reference. **(D)** Flow cytometry analysis of chimerism using peripheral blood from SS-chimera mice at 2 months after SS-to-wildtype bone marrow transplantation. A high percentage of CD45.2^+^ (SS donors) over CD45.1^+^ (recipients) marker were noticed in all examined immune cell types. Shown is the flow data of one representative SS-chimera (*n* = 3).

Next, we used bone marrow transplantation-derived SS- and AA-chimera mice for experiments (23), since it is more efficient to produce age- and sex-matched SS and AA mice by this method than interbreeding heterozygous Townes (SA) mice. We produced SS- and AA-chimera mice by transplanting donor bone marrow into two-month-old, x-ray conditioned male C57Bl/6 mice. At 2 months after bone marrow grafting, SS-chimera mice showed splenomegaly that is comparable or sometimes more severe than Townes SS mice (16) (Figure 3A). The red blood cells (RBCs) taken from AA-chimera were disc-shaped with a flatter, concave center under normoxia, and only exhibited a shrunken, circular shape after exposure to 1% oxygen for 1 h (Figure 3B). In contrast, the RBCs from SS-chimera showed a more irregular edge under normoxia and sickle-shaped morphology after exposure to 1% oxygen for 1 h (Figure 3B). Complete blood count (CBC) of peripheral blood showed an increase of white blood cells and reduction of hemoglobin (HGB), RBCs, hematocrit (HCT), platelets, and the mean corpuscular hemoglobin concentration (MCHC) in SS-chimera, when compared to wildtype and AA-chimera mice (Figure 3C).

Flow cytometry analysis of peripheral blood from the SS (CD45.2)-to-wildtype (CD45.1) congenic chimera showed >90% reconstitution of monocytes, neutrophils, and CD4 T cells plus near-80% chimerism in CD8 T cells (Figure 3D). These data support SS-chimera as an efficient way to produce SS-mutants with hematological anomalies similar to those in transgenic SS mice.

### SS-chimera mice show poorer recovery of CBF after MCA-targeted photothrombosis

**Figure 4:**
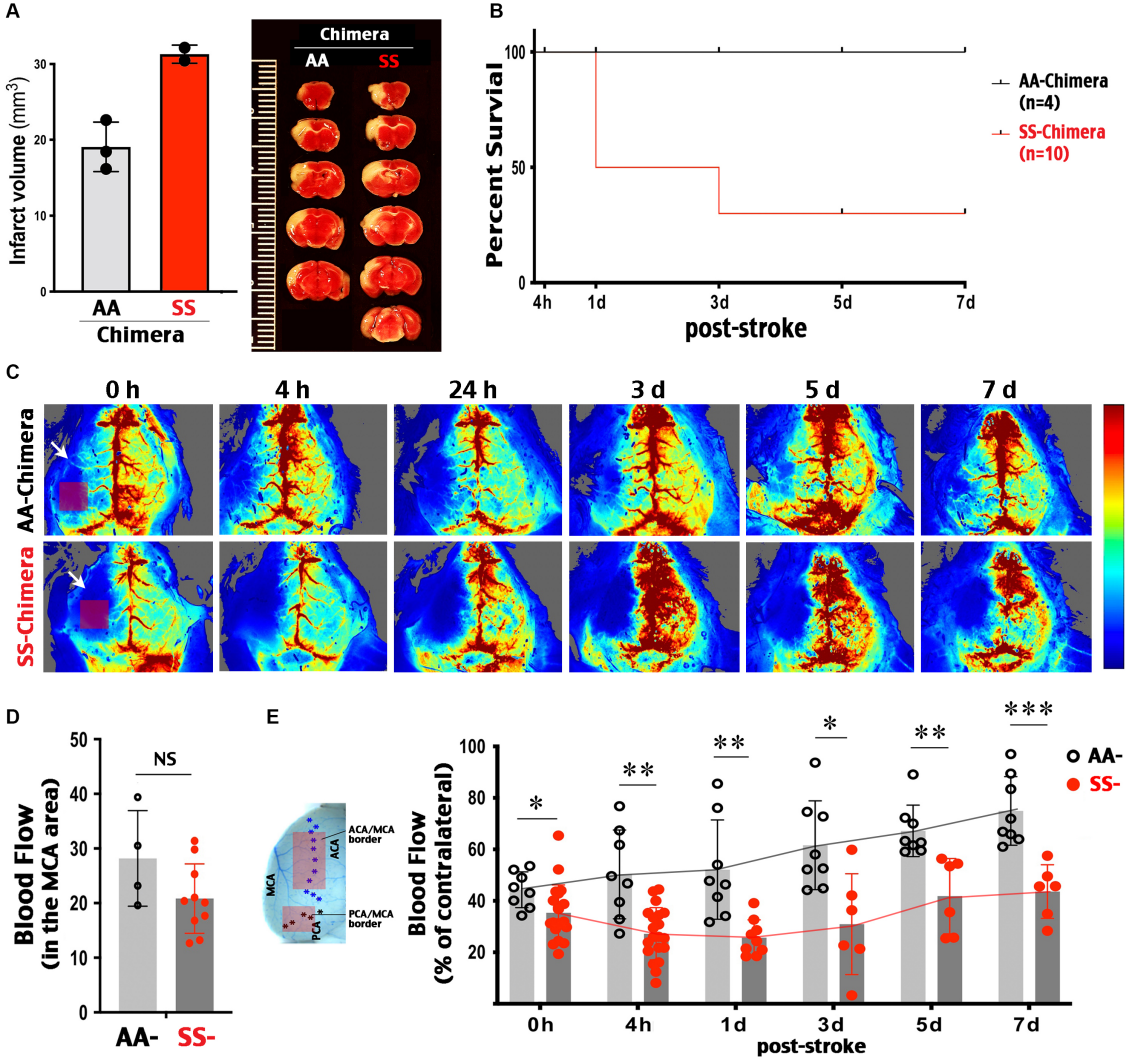
Post-stroke collateral circulation deficits in SS-chimera mice. **(A)** TTC stain at 3 days after MCA-targeted photothrombosis showed a trend of larger infarction in SS- than AA-chimera. SS chimera also showed reduced survival after MCA photothrombosis. **(B)** SS-chimera (*n* = 10) showed higher mortality after MCA-targeted photothrombosis compared to AA-chimera (*n* = 4). **(C)** Representative LSCI images showed attenuated CBF recovery in SS-chimera for up to 7 days after MCA-photothrombosis, when compared to AA-chimera. Arrow indicates the site of MCA-targeted photoactivation. Rectangle indicates the downstream MCA territory where the blood flow was measured acutely after photothrombosis to assess the degree of ischemia. **(D)** Quantification showed a comparable level of blood flow reduction in the MCA area acutely after photothrombosis between AA- (*n* = 4) and SS-chimera (*n* = 10). **(E)** Locations in the ACA-MCA and MCA-PCA borders (rectangles) where the LSCI values at both hemispheres were quantified to compare post-stroke collateral circulation. Quantification of the ipsilateral to contralateral LSCI ratio in the ACA-MCA and MCA-PCA border areas showed poorer recovery of CBF in SS-chimera, compared to AA-chimera, at all examined time-points after MCA-stroke. Every data point was the average of blood flow signals over 5 min in each mouse in the ACA-MCA or MCA-PCA border region, as indicated. * indicates *p* < 0.05, ** indicates *p* < 0.01, and *** indicates *p* < 0.001 by Mann–Whitney test.

Similar to transgenic Townes SS mice, SS-chimera showed enlarged infarction after the MCA-targeted photothrombosis (Figure 4A) and a reduced survival rate (30% at 7 d post-stroke, *n* = 10) compared to 100% survival of AA-chimera (*n* = 4) (Figure 4B).

LSCI was used to compare the changes of CBF between the MCA-targeted ipsilateral cortex and the contralateral cortex. We measured the percentage of blood flow in contralateral cortex because the intensity of LSCI signals is influenced by the skull thickness and hemoglobin concentrations (22), which is lower in SS than AA-chimera mice (Figure 3C). This analysis showed that MCA-targeted photothrombosis produced similar levels of blood flow reduction in the downstream MCA territory between SS- and AA-chimera mice (Figures 4C,D). Of note, photoactivation was targeted at the proximal MCA branch underneath the squamosal bones, while the blood flow of MCA-territory was imaged over the parietal cortex (indicated by arrow and square in Figure 4C, 0 h). Thus, the reduction of blood flow is due to occlusion of the upstream MCA proximal branch, but not direct photoactivation in the cortex as in some versions of photothrombosis.

Longitudinal LSCI tracking showed gradual recovery of blood flow in ipsilateral cortex over 7 days after MCA-photothrombrosis in AA-chimera mice (Figure 4C, upper row). In contrast, there was no sign of recanalization at the proximal MCA branch leading to recovery blood flow in the core of MCA territory in either SS- or AA-chimera mice during the entire 7 d period after photothrombosis (Figure 4C). When the MCA-anterior cerebral artery (ACA) and the MCA-posterior cerebral artery (PCA) border area was measured, the regional blood flow improved from 44.3 to 78% of contralateral values in AA chimera (Figure 4E). In contrast, the blood flow in the MCA-ACA and MCA-PCA border regions was consistently lower in SS- than AA-chimera mice from 0 h (*p* < 0.05) to 4 h (*p* < 0.01), 1 d (*p* < 0.01), 3 d (*p* < 0.05), 5 d (*p* < 0.01) and 7 d post-stroke (*p* < 0.001 by Mann–Whitney test) and only recovered from 35.4% of contralateral values at 0 h to 43.5% of contralateral values at 7 days (Figures 4C,E). These results suggest that SS chimera had poorer collateral circulation both acute and the secondary recovery phase after MCA-photothrombosis.

## Discussion

The causes for high incidence and poorer outcomes of ischemic stroke in SCA patients are complex and multifaceted (13). The known causes include anemia, stress-induced erythrocyte sickling, faster thrombus formation, chronic vasculopathy, and sensitivity to transient hypoxia-ischemia (17, 26–28). While anemia is a common risk factor for cerebrovascular complications, the presence of inflammatory or proliferative-occlusive vasculopathy in SCA is unique among hemoglobinopathy and contributes greatly to the high incidence of stroke in this population (28).

**Figure 5:**
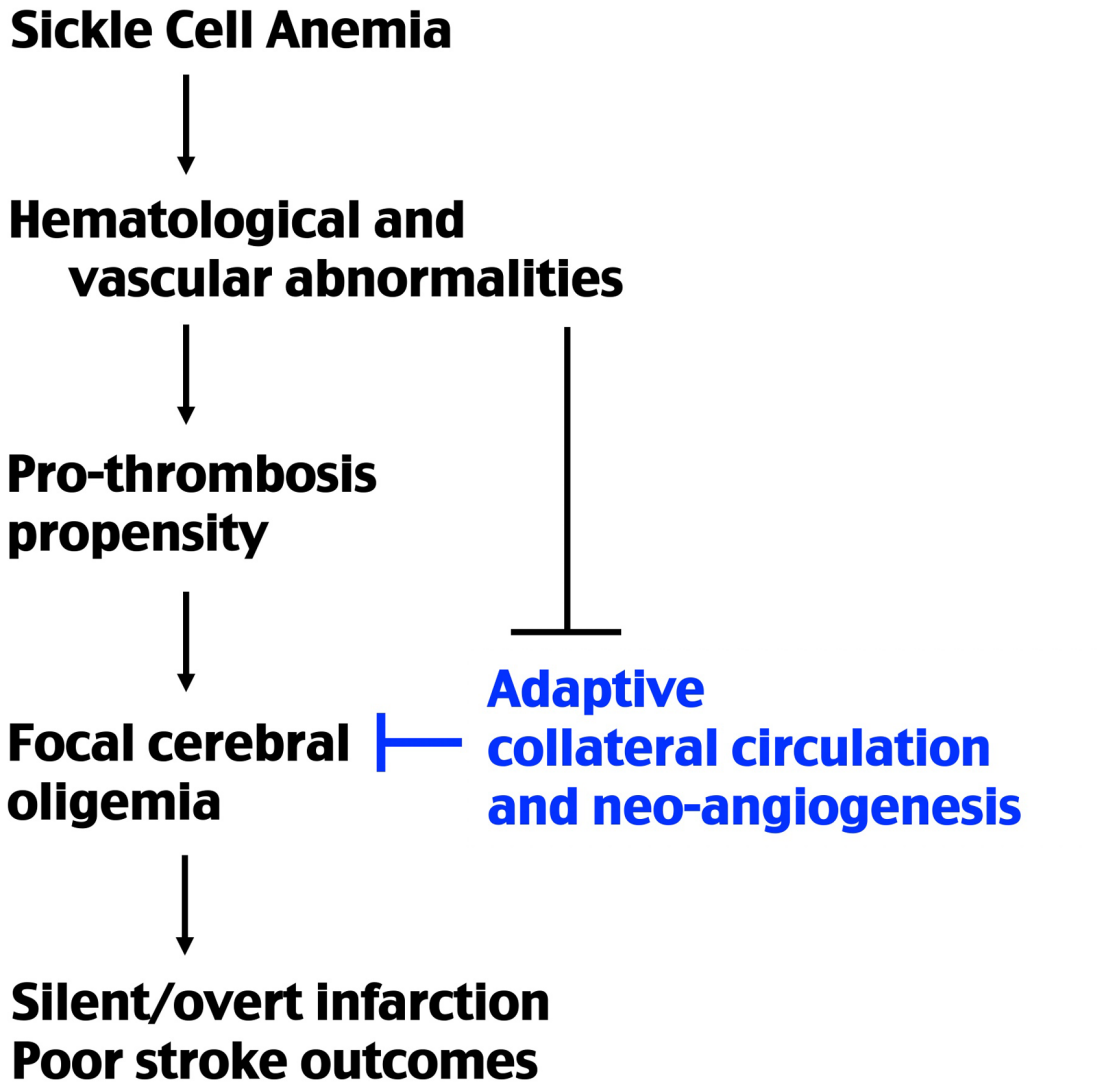
Schematic of the causes of stroke propensity and poorer outcomes in SCA. Previous studies showed that SCA causes a higher pro-thrombosis propensity under ischemic–hypoxic condition. The present study suggests that SCA also impairs post-ischemia redistribution of collateral blood flow and neo-collateral vessel formations. The combination of these factors produces a higher incidence of stroke and poorer outcomes in SCA patients.

Accordingly, transgenic sickle mice are more sensitive to hypoxia/ischemia-induced thrombosis than mouse models of β-thalassemia, despite similar levels of anemia (17, 29). Moreover, sickle mice develop high mortality and larger infarction after MCA-targeted photothrombosis, which only induces limited infarction and is usually non-lethal in wildtype mice, as shown in our results and previous studies (17). These findings suggest that SCA has unique pathophysiology that not only increases the stroke propensity, but also impairs post-stroke adaptation. Specifically, SS chimera mice showed reduced endothelial cell proliferation and attenuated CBF recovery for 7 d after MCA-photothrombosis, similar to impaired collateral vessel formation after limb ischemia in a previous report (19). These findings collectively suggest that deficits in collateral circulation may contribute to poorer stroke outcomes and possibly the formation of silent cerebral infarct in SCA patients (Figure 5). Precise mechanisms of SCA-induced deficits in post-stroke collateral circulation remain unclear, and here we discuss some possibilities based on the literature of this topic.

### Acute recruitment of collateral circulation after stroke

The sources of collateral circulation to cerebral cortex include extracranial-to-intracranial anastomoses, communicating arteries in the circle of Willis, and the leptomeningeal anastomoses between the ACA, MCA, PCA, and superior cerebellar artery branches (2). Among them, the leptomeningeal anastomoses between ACA, MCA, and PCA branches have the best evidence for stroke protection effects to date. First, live imaging of cortical surface micro-vessels revealed an acute and robust redistribution in blood flow after vascular occlusion (5). Second, the numbers of leptomeningeal anastomoses between the ACA, MCA, and PCA trees vary considerably among 21 inbreed mouse strain but correspond to their susceptibility to experimental stroke (30). Third, aging and diabetes, two major risk factors for poor stroke outcomes, show lesser leptomeningeal collateral flow between the ACA, MCA, and PCA branches in rodent models (10, 11). Finally, clinical trials indicated that strong ACA-to-MCA collateral circulation at the time of acute stroke predicts favorable outcomes for thrombolytic or thrombectomy therapy (8, 9). These findings suggest intrinsic adaptation in cerebral perfusion that can quickly redirect blood flow toward the ischemic area, but this protective mechanism is impaired by the pathologies in aging, diabetes, or sickle cell anemia.

The cellular mechanisms for this vascular adaptation remain poorly understood, but the endothelial nitric oxide synthase (eNOS) and sphingosine 1-phospahte (S1P) signaling pathways may be important mediators (6, 7). It has been shown that the phosphorylation state of eNOS modulates vascular reactivity and the outcomes of cerebral ischemia, whereas eNOS deficiency causes cortical collateral vessel rarefaction (31, 32). Gene-deletion experiments also showed that endothelial S1P receptor-1 (S1P_1_) signaling opposes infarct expansion in ischemic stroke (33). It has been suggested that shear stress after vessel obstruction stimulates S1P_1_ on endothelial cells, which in turn activates the eNOS signaling for local vascular dilation (34). It is also possible that S1P_1_ activation in the capillaries may propel retrograde signals to relax the pericytes and smooth muscle cells in the arterioles and produce broad vasodilation (35). Since there is a higher level of S1P in the blood of mice and humans with SCA (36), future studies are warranted to test whether the quantity or quality of S1P receptors are diminished by SCA or other stroke risk factors, such as aging and diabetes.

To harness the potential of these protective responses to ischemia, systemic application of S1P_1_ agonist (e.g., SEW2871) or adropin peptides are promising approaches (34, 37). Adropin is particularly intriguing, since it is a physiological regulator of glucose and lipid metabolism and the only available agent to enhance the eNOS signaling to date (38, 39). Further, age-dependent decrease in adropin is associated with reduced levels of eNOS, while intravenous application of recombinant adropin peptides opposes ischemic stroke in an eNOS-dependent manner (37, 40). Future studies are merited to test whether S1P_1_ activation or adropin treatment provides ischemia protection through up-regulation of acute post-stroke collateral circulation.

### Collateral remodeling and neo-collateral formation after stroke

Several lines of evidence suggest that after acute post-stroke collateral recruitment, peri-infarct vessels undergo remodeling and neo-collateral formation to foster brain tissue repair (4). This process is likely modulated by the interactions between vascular endothelium and myeloid infiltrates, including CCR2^+^ monocytes/macrophages and N2-type neutrophils (41–43). Further, hypoxia increases the expression of *Vegf*, *Hif2α*, and *Rabep2* (the gene that underlies differential mouse strain vulnerability in stroke) and induces de-novo formation of cerebral collaterals (44). It is conceivable that SCA may modify the properties of vascular endothelium and myeloid cells, leading to attenuated collateral remodeling and/or neo-collateral formation. Although post-stroke injection of vascular endothelial growth factor (VEGF) seems a reasonable strategy to stimulate neo-collateral formation, VEGF is also known as vascular permeability factor (VPF) and induces blood–brain-barrier (BBB) leakage in the ischemic brain (45). Thus, post-stroke VEGF treatment may be counterproductive, a concern that is heightened by the lack of reporting positive results in such clinical trials (ClinicalTrials.gov identifiers: NCT02157896 and NCT00134433) (46). In view of the caveats of VEGF treatment, the recently introduced engineered Wnt7a (eWnt7a) with potent and selective activation of Reck/Gpr124 may be better therapeutics to enhance post-stroke collateral circulation, because Wnt7a-activation simultaneously induces angiogenesis and the maturation of BBB (47, 48).

### Limitations of the present study

The biggest limitation of this report is the lack of detailed analytic or intervention studies to test the roles of these putative signaling pathways. In addition, given the small sample size, we were not able to stratify the animals to test the impacts of anemia severity and RBC morphology, such as red cell distribution width (RDW) on collateral circulation. Future studies are needed to compare the blood flow at the individual cerebral vessel level, since a recent study reported more frequent flow reversals in Townes SS mutants than AA mice (49). Finally, since our whole-body X-irradiation replaced the brain microglia with those carrying the hemoglobin SS mutations in SS-chimera (50), future studies are warranted to compare the responses of collateral circulation in SS-chimera generated by head-shielded X-irradiation.

## Conclusion

In closing, our results suggest that impairment in post-stroke collateral circulation is an important contributor to greater stroke vulnerability in SCA. Through the comparison of SS and wildtype mice, one could identify the mechanisms regulating post-stroke collateral circulation to develop therapeutic strategies. Such treatments may benefit SCA patients and those with other risk factors for poorer stroke outcomes.

## Data availability statement

The raw data supporting the conclusions of this article will be made available by the authors, without undue reservation.

## Ethics statement

The animal study was approved by the University of Virginia IACUC. The study was conducted in accordance with the local legislation and institutional requirements.

## Author contributions

C-YK and Y-YS conceived the research idea and experimental plan. EB, C-WC, C-MC, and Y-YS performed the experiments. C-YK, EB, and Y-YS wrote the manuscript. All authors contributed to the article and approved the submitted version.

## Funding

This work was supported by the National Institute of Health grants (NS108763, NS125788, NS125677, NS127392, and HD109025 to C-YK and NS106592 to Y-YS); the Ministry of Science and Technology (MOST 111-2320-B-110 -003 -MY2 to Y-YS); the Kaohsiung Veterans General Hospital (VGHNSU 111-012 to Y-YS); and the National Sun Yat-sen University-Kaohsiung Medical University Joint Research Project (NSYSUKMU 111-P15 and 112-P26 to Y-YS).

## Conflict of interest

The authors declare that the research was conducted in the absence of any commercial or financial relationships that could be construed as a potential conflict of interest.

## Publisher’s note

All claims expressed in this article are solely those of the authors and do not necessarily represent those of their affiliated organizations, or those of the publisher, the editors and the reviewers. Any product that may be evaluated in this article, or claim that may be made by its manufacturer, is not guaranteed or endorsed by the publisher.
